# Sensitive Quantification of Liensinine Alkaloid Using a HPLC-MS/MS Method and Its Application in Microvolume Rat Plasma

**DOI:** 10.1155/2021/6629579

**Published:** 2021-02-26

**Authors:** Fen Wei, Xilan Gou, Xiao Xu, Sicen Wang, Tao Bao

**Affiliations:** ^1^School of Pharmacy, Health Science Center, Xi'an Jiaotong University, Xi'an 710061, China; ^2^Shaanxi Engineering Research Center of Cardiovascular Drugs Screening & Analysis, Xi'an 710061, China

## Abstract

Liensinine, an important alkaloid in lotus seed, exhibits multiple functions such as anti-AIDS, anticancer, antidepressant, and antihypertensive properties. In this study, a highly sensitive HPLC-MS/MS method was developed and validated for the quantification of liensinine in microvolume rat plasma as low as 45 *μ*L. Chromatographic separation was carried out using a reverse-phase Gemini-C18 column (100 mm × 3 mm i.d. × 5 *μ*m), and mass selective detection using multiple reaction monitoring was attained using an electrospray ionization source, which operated in the positive mode. Dauricine was used as the internal standard. The precursor-to-product ion transition m/z 611.15 > 206.10 was selected for the detection of liensinine; m/z 625.25 > 206.10 was used for the detection of dauricine. The developed method is linear over the concentration range of 0.05–1000 ng/mL with an excellent coefficient of determination (*R*^2^ = 0.991). The recoveries ranged from 92.57% to 95.88% at three quality control levels. Intraday and interday precision and accuracy are less than 12.2% and 6.59%, respectively. The lower limit of quantification (LLOQ) is 0.05 ng/mL. The matrix effect was insignificant and acceptable. The validated method was successfully applied to the pharmacokinetic study of liensinine in rats. This method can be used for *in vivo* studies as well as quality control of traditional Chinese medicines and herbal tea containing liensinine alkaloid.

## 1. Introduction

*Lotus plumule* is the green embryo of a lotus seed located between two cotyledons [1]. In China, it is commonly consumed as a kind of tea in summer. *L. plumule* is widely used as an anti-inflammatory, antidepressant, cooling, and astringent agent in Southeast Asia and China [2]. Various active components have been separated and characterized from lotus including alkaloids, fatty oils, flavonoids, triterpenoids, and vitamins [3–6]. Alkaloids are the major constituents of lotus [7]. Similar to other bisbenzylisoquinoline alkaloids such as isoliensinine and neferine, liensinine also exhibits anti-HIV [8], antiarrhythmic [9, 10], anti-inflammatory [11, 12], antipyretic [13], anticancer [14], antidepressant [15, 16], and antihypertensive [17] properties.

To ensure the safety and efficacy of liensinine, an accurate, valid, and highly sensitive detection method is needed. Various methods including HPLC [18, 19], thin-layer chromatography (TLC) scanning [20], high-speed countercurrent chromatography (HSCCC) [21, 22], LC-MS [23–25], and HPLC-TOF-MS [26] have been developed for the determination of liensinine alkaloid in plant extracts and biological samples. These methods are either less sensitive or require large volumes of plasma samples. Moreover, the sample preparation techniques are also inefficient; for example, only centrifugation is used for sample preparation [25].

In this study, we developed a highly sensitive and fully validated HPLC-MS/MS method for the detection and quantification of liensinine alkaloid. The method offers a small turnaround time for analysis and high sensitivity using a simple liquid–liquid extraction of microvolume rat plasma (45 *μ*L). The effect of sample preparation on recovery and the matrix effect is demonstrated by comparing two different extraction procedures, that is, protein precipitation (PPT) and methyl *tert*-butyl ether (MTBE) liquid-liquid extraction (LLE). Compared with PPT, MTBE LLE provided the cleanest samples with a higher recovery and reduced matrix effect. A comparative study (high pH vs. low pH mobile phases) using two different reverse-phase columns (pH stable Gemini-C18 vs. normal Sunfire-C18) was also conducted to evaluate the effect of pH on the sensitivity of liensinine alkaloid. Compared with acidic conditions (pH 2.5), a higher sensitivity was observed in high pH (10) conditions. The lower limit of quantification (LLOQ) is 0.05 ng/mL, 100 times lower than the previously reported LLOQ [23, 25]. The developed method can be used for *in vivo* studies and quality control (QC) of traditional Chinese medicines and herbal tea containing liensinine alkaloid.

## 2. Materials and Methods

### 2.1. Reagents and Standards

The following chemicals and reagents were used: liensinine alkaloid was purchased from Tianjin Shilan Tech., China. The internal standard (IS) dauricine was purchased from Beijing Beina Chuanglian Biotechnology Institute (Beijing, China). HPLC-grade methanol and acetonitrile (ACN) were purchased from Thermo Fisher Scientific (Pittsburgh, PA, USA). Mass-grade formic acid and ammonium hydrogen carbonate (NH_4_HCO_3_) were purchased from Sigma-Aldrich (Switzerland). MTBE was purchased from Shanghai Richjoint Chemical Reagents (Shanghai, China). Water was produced using a MK-459 Millipore Milli-Q Plus ultrapure water system. Plasma was separated from blood collected by retro-orbital bleeding of rats.

### 2.2. Instrumentation and HPLC-MS/MS Conditions

A Shimadzu (Kyoto, Japan) triple quadrupole tandem mass spectrometer LC-MS 8040 equipped with electrospray ionization (ESI) was used for analysis. The equipment consists of two Nexera LC-20AD pumps, a CTO-20AC column oven, a DGU-20A degasser (Shimadzu Corporation, Kyoto, Japan), a SIL-20AXR autosampler, and a 20A communication bus module. The LC-MS/MS was interfaced and controlled using the computer software “Lab Solutions^®^ version 5.72” (Shimadzu Corporation, Kyoto, Japan). A Phenomenex^®^ Gemini RP-C18 column Torrance, CA, USA (100 mm × 3 mm i.d. × 5 *μ*m) with pH stability range of 1–12 and Waters^®^ Sunfire-C18 column Milford, USA (150 × 2.1 mm i.d. × 3.5 *μ*m) with pH stability range of 2–8 at 40 °C were used. LC mobile phase A consists of 10 mM ammonium hydrogen carbonate buffer (adjusted to pH 10 with aqueous ammonium hydroxide) and mobile phase B consists of 50 : 50 (v/v) mixture of ACN-methanol at a flow rate of 0.4 mL/min under a simple binary gradient time program. The gradient time program was used as follows: an initial short isocratic portion of 10% B for 0.3 min, followed by a linear gradient portion where B was increased from 10% to 95% over 1 min, isocratic elution of 95% B for the next 3 min, and then the column was returned to its initial conditions within 1 min. The flow rate was increased from 0.4 mL/min to 1.0 mL/min at time 3.0–3.5 min, held at this flow rate for the next 0.5 min, and returned to the initial flow rate (0.4 mL/min) at 4.5 min, and the column was reequilibrated for the analysis of next sample. The total run time was 7.0 min, including the reequilibration of the column. This strategy was useful to remove strongly retained matrix components on the RP column, thus increasing the column life and ensuring that there is a slight carryover effect in the subsequent runs.

The mass spectrometer was operated in the positive electrospray ionization mode with multiple reaction monitoring (MRM) for both target analyte (liensinine) and IS. The mass spectrometric parameters were optimized to acquire the highest MRM signals. The parent-to-product ion pair m/z 611.15 > 206.10 was used for the MRM of liensinine, and m/z 625.25 > 206.10 was used for IS. An online six-port two-position valve was either used to introduce the effluent from the LC system to the mass spectrometer during the peak elution time, that is, from 2.25 min to 3 min, or diverted to drain before and after the peak elution time.

The interface parameters were also optimized to produce the maximum intensity of ions. The optimized interface parameters are as follows: nebulizing gas flow of 3.0 L/min (N_2_, purity >99.999%), desolvation line (DL) temperature of 280°C, heat block temperature of 400°C, drying gas flow of 15.0 L/min, interface voltage of 4.5 kV, interface current of 0.3 *μ*A, detector voltage of 1.88 kV, CID gas (He, purity >99.999%), and pressure of 230 kPa. Collision energy (CE) for liensinine and IS was 36.0 eV and 39.0 eV, respectively.

### 2.3. Preparation of Solutions

**Figure 1:**
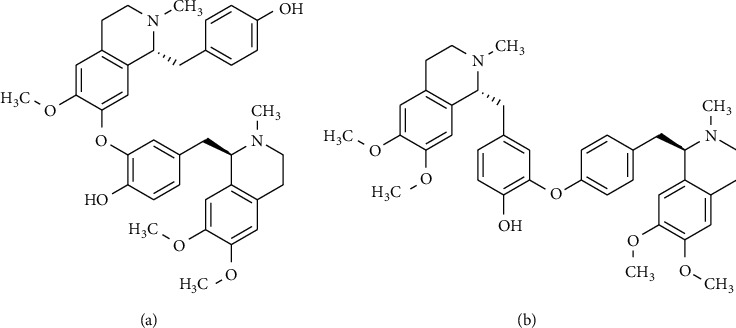
Chemical structure of (a) liensinine and (b) dauricine.

A stock solution of liensinine (Figure 1(a)) was prepared by dissolving 1 mg/mL in methanol. The stock solution was stored at −20°C in the dark. Working standard solutions (0.5, 1, 5, 50, 500, 1000, 3000, 5000, and 10000 ng/mL) in methanol-water (5 : 95, v/v) were prepared from the stock solution. Calibration standard solutions (0.05, 0.1, 0.5, 5, 50, 100, 300, 500, and 1000 ng/mL) were prepared by spiking the drug-free rat plasma (45 *μ*L) with 5 *μ*L of the corresponding working solution. Three QC samples of 0.05 (low QC), 5.0 (medium QC), and 750 ng/mL (high QC) were prepared to determine the matrix effects, precision, accuracy, recovery, and stability of the method. The QC samples were prepared in blank plasma (45 *μ*L) by spiking with 5 *μ*L of the corresponding working solutions (0.5 ng/mL, 50 ng/mL, and 7500 ng/mL), respectively. Similarly, a dauricine (Figure 1(b)) stock solution was prepared at a concentration of 1 mg/mL by dissolving in methanol. This stock solution was diluted with methanol-water (5 : 95, v/v) to produce a final concentration of 20 ng/mL.

### 2.4. Sample Extraction

For drug extraction, 50 *μ*L of spiked sample and 50 *μ*L of IS (20 ng/mL) solution were mixed in a prelabeled centrifuge tube by vortex mixing for 30 s. The mixture was extracted with 350 *μ*L of MTBE by vortex mixing at a high speed for 2 min and then centrifuged at 14,500 g for 10 min. The upper organic layer was transferred to another prelabeled 1.5 mL Eppendorf tube. The supernatant was dried under a stream of N_2_ at 45°C, and the residue was reconstituted in 200 *μ*L methanol-water (5 : 95 v/v). This reconstituted solution was sonicated for 5 min in an ultrasonicator at room temperature, then filtered (0.22 *μ*m membrane filter), and transferred to autosampler vials. Finally, 5 *μ*L was injected into the HPLC-MS/MS system for analysis.

### 2.5. Method Validation

The method was validated for specificity, calibration curve, sensitivity, recovery, carryover, matrix effect, precision, and accuracy (intraday and interday).

Specificity was assessed by analyzing the blank plasma obtained from six different rats and comparing them with blank plasma samples spiked and extracted at the LLOQ.

The linearity of the method was estimated in the concentration range of 0.05 ng/mL to 1000 ng/mL by analyzing nine calibration standards. The mean peak area ratio of analyte/IS (*y*-axis) was plotted against analyte standard concentration (*x*-axis) to construct a calibration curve. Least square linear regression was used to obtain a regression equation (*y* = *mx* + *b*) to determine the coefficient of determination (*R*^2^), where *y* is the area ratio of analyte/IS; *m* is the slope; *x* is the nominal concentration of analyte; and *b* is the *y*-intercept. A signal-to-noise ratio (S/N) of 3 was used to determine the limit of detection (LOD), and S/N 10 was used to determine LLOQ.

Recovery of analyte from the matrix was determined at three QC levels. The mean peak area ratios of analyte/IS obtained from the prespiked and extracted samples were compared with those of the same concentration of postextracted and spiked samples (*n* = 5). As per acceptance criteria, the recovery of analyte and IS did not need to be 100% but should be reproducible, precise, and persistent.

The matrix effect was evaluated by comparing the mean peak area ratio of analyte/IS (postextracted and spiked samples) with those of the same concentration of standard solutions (*n* = 5). As per acceptance criteria, the matrix effect should not be >15% compared to the standard solutions.

The intraday and interday precision and accuracy were determined at three QC levels, on the same day, and on three consecutive days. As per acceptance criteria, precisions and accuracy should not be >15% of actual concentration except for LLOQ (≤20%).

Stability studies were performed at four QC levels, namely, 12 hours at ambient temperature (short period), in autosampler conditions for 24 h, after three freeze-thaw cycles, and at −20°C for one month (long period). The acceptance criterion was 90–110% in comparison to freshly prepared solutions.

### 2.6. Application to a Pharmacokinetic Study

Healthy male Sprague Dawley rats (*n* = 15) weighing 220 g ± 20 g were obtained from the experimental animal center, Xian Jiaotong University, China. Prior to starting the experiment, the rats were housed (under control environmental conditions with free access to food, water, and 12 h dark/light cycles). Food was withheld for 12 h after dose and resumed 4 h after the start of sampling. Five days of acclimation period was given to rats to adjust in the new environmental conditions. After 12 h dark/light cycle and fasting for 12 h, three groups of rats received a single oral dose of 5, 12.5, and 25 mg/kg of liensinine by oral gavage. Blood was collected from rats using retro-orbital bleeding method. The rats were mildly anesthetized with diethyl ether to give minimum distress. A series of blood sampling was performed at the intervals of 0.00, 0.08, 0.25, 0.5, 1, 3, 6, 9, 12, and 24 h from each rat. The blood was collected in 1.5 mL Eppendorf tubes (treated with 0.5% heparin in normal saline). The blood samples were centrifuged (14,000 g at 4°C for 10 min) immediately to obtain plasma (90 *μ*L). The plasma samples were stored at −20°C in the dark until analysis.

### 2.7. Statistical Analysis

The Shimadzu “Lab Solutions^®^ version 5.72” software was used for peak area integration. Least square linear regression was used to generate calibration curve data. GraphPad prism 5.0 was used to generate plasma-time concentration curves. Liensinine plasma concentration versus the time data for each group of rats was analyzed using Drug and Statistics software (DAS, Version 3.0, Shanghai BioGuider Medicinal Technology, China).

## 3. Results and Discussion

### 3.1. Development and Optimization of HPLC-MS/MS Conditions

**Figure 2:**
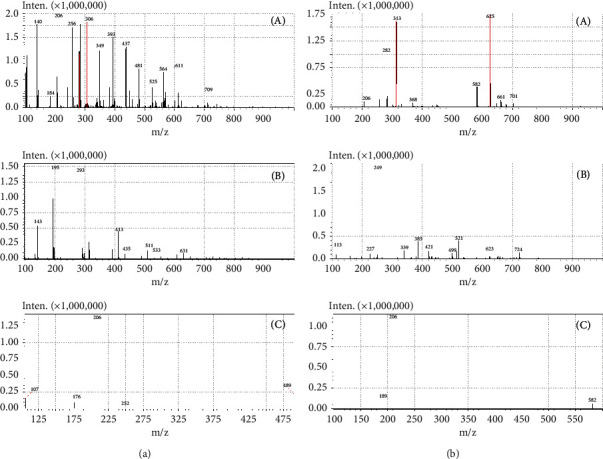
Full-scan mass spectrum of (a) liensinine and (b) dauricine: (A) ESI positive, (B) ESI negative, and (C) product ion scan.

Full-scan mass (100–1000 Da) spectra, that is, ESI positive and ESI negative for liensinine and IS, are shown in Figure 2(a). The best ionization mode for liensinine and IS is ESI^+^. As protonated [*M* + *H*]^+^ precursor ions were produced for liensinine (m/z 611.15) and IS (m/z 625.25), the precursor ions [*M* + *H*]^+^ were fragmented in the tandem mode by collision-induced dissociation (CID) to produce the corresponding product ions (Figure 2(b)). Parent-to-product ion mass transition 611.15 > 206.10 was selected for the detection of liensinine and m/z 625.25 > 206.10 for IS.

**Figure 3:**
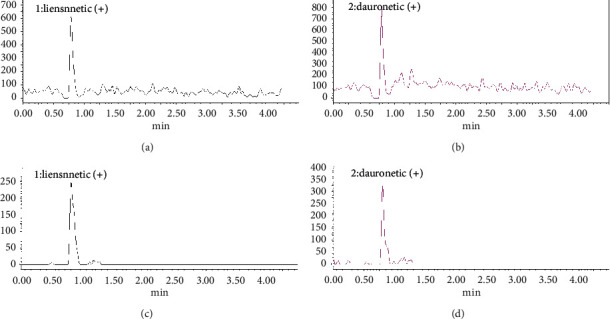
Chromatogram of (a) liensinine 10 ng/mL and (b) dauricine 20 ng/mL on a Sunfire-C18 column. (c) Liensinine 10 ng/mL and (d) dauricine 20 ng/mL on a Gemini-C18 column at low pH conditions (0.1% formic acid pH 2.5).

During the development of LC method, different mobile phases were evaluated on a trial and error base. These included ACN, methanol, ACN-water mixture, methanol-water mixture with 0.1% formic acid (pH 2.5) and without formic acid, or 10 mM NH_4_HCO_3_ buffer (pH 10). Liensinine and IS are basic in nature and mainly exist in the ionized form at a low pH (0.1% formic acid, pH 2.5). Therefore, peaks with short retention time (less than 1 min) were observed, and less intensity was observed on both columns (Waters Sunfire RP-C18, 150 mm × 2.1 mm, i.d. × 3.5 *μ*m, and Gemini RP-C18, 100 mm × 3.0 mm i.d. × 5 *μ*m (Figures 3(a) and 3(c)). Mobile phase A was 0.1% formic acid (pH 2.5), and mobile phase B was an ACN/methanol (50 : 50 v/v) mixture. The flow rate was 0.4 mL/min. These results (retention time and intensity of analyte) agree with those obtained in previous studies [23–25] under the most commonly used 0.1% formic acid conditions. If the retention time is short, early elution of hydrophilic matrix components may interfere with the ionization process of the analyte. Because these hydrophilic matrix components are poorly retained on the RP-C18 column, they are coeluted with the analyte(s) [27].

**Figure 4:**
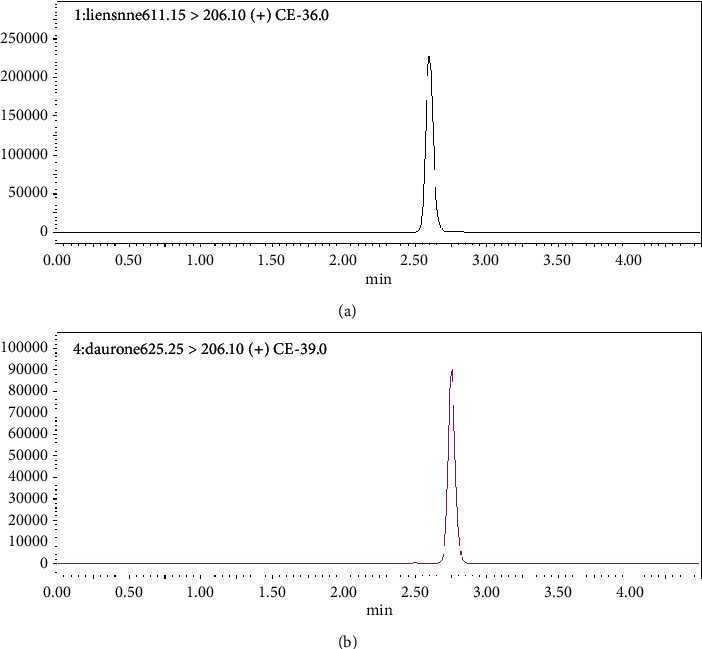
Chromatogram of (a) liensinine 10 ng/mL, retention time 2.58 min; (b) dauricine (IS) 20 ng/mL, retention time 2.75 min, on Gemini-C18 column at high pH conditions (10 mM NH4HCO3 buffer pH 10).

Basic analytes mostly exist in the unionized form at high pH, two units higher than their pKa value. In the high pH mobile phase, basic compounds result in longer retention, good peak shapes, and excellent chromatographic efficiencies [28, 29]. Ammonium ions are volatile in nature and compatible with a mass spectrometer; thus, 10 mM ammonium hydrogen carbonate buffer at pH 10 was used as the mobile phase A. Mobile phase B is a mixture of ACN-methanol (50 : 50 v/v). High-intensity peaks, good resolution, longer retention (liensinine 2.58 min and IS 2.75 min), and symmetry were observed on a Gemini RP-C18, 100 mm × 3.0 mm i.d. × 5 *μ*m column (Figure 4). The Waters Sunfire RP-C18 column was not used at a high pH due to the possibility of silica dissolution. Hence, high pH conditions ensured a higher sensitivity in contrast to low pH conditions [2, 23–25].

### 3.2. Optimization of Sample Preparation

During the optimization of sample preparation, two different extraction procedures, that is, PPT and LLE, were carried out. Methanol and ACN were tested as protein precipitants. MTBE was used for LLE. For PPT, 100 *μ*L ACN was added to 50 *μ*L of spiked samples. The same procedure was used for PPT with methanol. For MTBE LLE, MTBE (350 *μ*L) was added to the spiked samples (50 *μ*L). The results of liensinine recoveries following PPT with double volume of ACN and methanol are comparable, as shown in Table 1. However, a pronounced matrix effect was observed when methanol was used as the extraction solvent, probably due to the high solubility of lipids in methanol. MTBE LLE produced the cleanest samples with high recovery, whereas a reduced recovery was observed when methanol was used for sample preparation. Therefore, the samples were extracted with MTBE LLE as described in Section 2.4.

### 3.3. Method Validation

**Figure 5:**
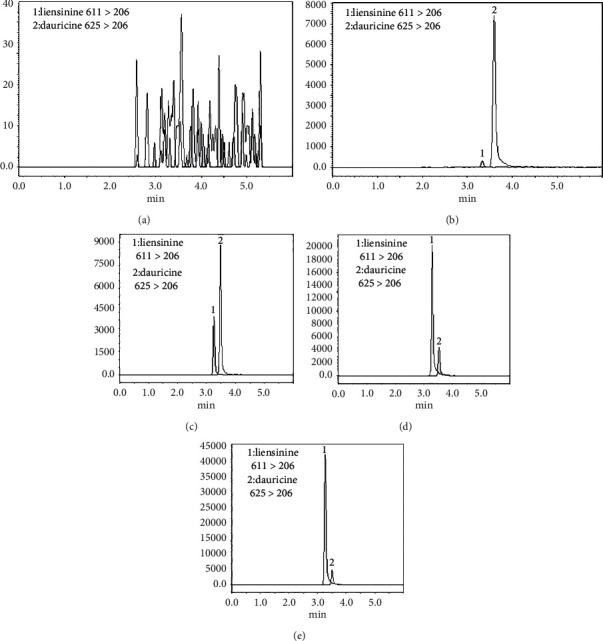
Representative MRM chromatograms of the liensinine and dauricine (IS) in rat plasma samples. (a) A blank plasma sample. (b) blank plasma sample spiked with liensinine in LLOQ and IS (20 ng/mL). (c), (d), (e) A rat plasma sample taken 3 h after oral administration of liensinine 5.0 mg/kg, 12.5 mg/kg, 25.0 mg/kg in rats; (1) liensinine and (2) dauricine (IS) (20 ng/mL).

Article selectivity of the method was evaluated by comparing the chromatograms of blank samples from six different rats with the chromatograms of their corresponding spiked samples. Typical chromatograms of blank samples (A), blank samples spiked with the analytes at LLOQ and IS (B), and the biological samples obtained from rats after 3 h for plasma oral administration of 5.0 mg/kg (C), 12.5 mg/kg (D), and 25.0 mg/kg (E) liensinine in rats (C) are shown in Figure 5. No endogenous interference was detected at the retention time of analyte and IS, indicating that the article selectivity of the method was satisfactory.

The developed method was linear at a concentration range of 0.05 ng/mL to 1000 ng/mL. The coefficient of determination (*R*^2^) was 0.9913. The regression equation was found to be *y* = 0.1255*x*−0.8251. The lowest concentration of calibration curve of 0.05 ng/mL determined at S/N of 10 represents the sensitivity of the method (LLOQ), whereas the LOD is 0.001 ng/mL at S/N = 3. Previously reported LLOQ values are 167 ng/mL [2], 36 ng/mL [18], 5 ng/mL [23], 10 ng/mL [24], and 5 ng/mL [25]. Therefore, the developed method is 100 times more sensitive compared to the lowest value (5 ng/mL) reported so far. Highly sensitive methods are helpful in understanding the time course of drugs in the body. As the drug concentration in the body can be monitored for several half-lives, this is an important parameter for assessing the degree of accumulation (toxicity) of a drug in the body.

Furthermore, blood samples were taken at several timepoints to determine the preclinical pharmacokinetic parameters in rodents. Taking large blood volumes often disturbs the normal blood physiology, producing false results [30]. Many methods have been developed for the determination of liensinine alkaloid in biological samples using plasma samples of more than 100 *μ*L [2, 18, 23, 25]. Our method uses only 45 *μ*L of rat plasma.

The recovery (Table 2) of the analyte after extraction with MTBE LLE showed that the extraction method is highly efficient in recovering the target analyte from the matrix. The recovery of analyte at three QC levels ranged from 92.57% to 95.88%.

Carryover was assessed during method validation by injecting blank samples after calibration standard at the upper limit of quantification. The carryover effect was calculated using the regression equation. The results show that the carryover in the blank sample following the high concentration standard was 4.94% (liensinine alkaloid) and 4.63% (IS), satisfying the criterion.

The matrix effect results (Table 3) indicate that the matrix effect is within acceptable limits, ranging from 93.26% to 106.47%. Insignificant suppression and enhancement were observed at MQC and HQC, respectively. However, both the suppression and enhancement were within the limits. The results of precision and accuracy (Table 4) show that the interday and intraday accuracies (% RE) are less than 6.59%, whereas the interday and intraday precisions (% RSD) ranged from 3.28% to 12.2%, respectively. The results of different stability evaluations are shown in Table 5, indicating that the samples were stable during the intended study period.

### 3.4. Pharmacokinetic Study

The validated method was successfully applied to the pharmacokinetic studies of liensinine in rat plasma after intragastric administration of 5, 12.5, and 25 mg/kg, respectively. The article sensitivity and selectivity of the method are appropriate for the analysis. The mean (±SD) plasma concentration-time profiles of the analyte after intragastric administration (*n* = 5) of liensinine at 5, 12.5, and 25 mg/kg were evaluated. The main pharmacokinetic parameters for liensinine were calculated using DAS V3.0 and noncompartmental analysis (Table 6). The main pharmacokinetic parameters for liensinine following intragastric administration [23–25] at 5 mg/kg were compared using DAS V3.0 and noncompartmental analysis (Table 7). After intragastric administration of liensinine, the plasma concentrations of liensinine decreased sharply within 3 h and then reached equilibrium at 12 h. The concentration-time data of liensinine in rat plasma best ﬁtted to the one-compartment open model. The AUC_0–∞_ and *C*_max_ values after intragastric administration show a significant decrease compared to the results of intravenous administration [23–25], indicating that liensinine had a lower bioavailability *in vivo.* The values of AUC_0*-t*_ and AUC_0–∞_ and *F* increased in a dose-dependent manner from 5 to 25 mg/kg. Taken together, this indicates that the Pk profile for liensinine is linear over the dose range used.

## 4. Conclusion

This study reports a highly sensitive and validated HPLC-MS/MS method for quantifying picograms of liensinine alkaloid in microvolume rat plasma. The LLOQ is 0.05 ng/mL, and the LOD is 0.001 ng/mL. The validated method is 100 times more sensitive than previously reported methods. This method can be used for in vivo studies as well as QC of traditional Chinese medicines and herbal tea containing liensinine alkaloid.

## Figures and Tables

**Table 1:** Liensinine and dauricine (IS) recoveries following extraction from rat plasma with three different solvents (*n* = 3).

Extraction solvent	Recovery (%)	
	Liensinine (100 ng/mL)	Dauricine (IS) (100 ng/mL)
Methanol	68.68 ± 6.12	71.54 ± 8.20
Acetonitrile	72.19 ± 4.78	65.44 ± 6.10
MTBE	96.42 ± 3.22	95.57 ± 2.72

**Table 2:** Recovery of liensinine at three QC levels.

Concentration (ng/mL)	Mean area ratio of prespiked and extracted (*n* *=* *5*)	Mean area ratio of postspiked and extracted (*n* *=* *5*)	Recovery (%)	RSD (%)
0.05 (LQC)	0.041308	0.044622	92.57	4.41
5.0 (MQC)	2.004632	2.102143	95.36	2.80
750 (HQC)	38.56806	40.22435	95.88	6.27

**Table 3:** Matrix effect of liensinine at three QC levels.

Concentration (ng/mL)	Mean area ratio of postspiked and extracted (*n* *=* *5*)	Mean area ratio of neat samples (*n* *=* *5*)	Matrix effect (%)	RSD (%)
0.05 (LQC)	0.044622	0.045278	98.60	13.91
5.0 (MQC)	2.095712	2.247012	93.26	11.09
750 (HQC)	40.22435	37.62342	106.47	4.79

**Table 4:** Intraday and interday precision and accuracy for liensinine at three QC levels.

Nominal concentration (ng/mL)	Intraday (*n* = 5)		Interday (*n* = 5)	
	RE (%)	RSD (%)	RE (%)	RSD (%)
0.05 (LQC)	5.0	9.40	6.59	12.2
5.0 (MQC)	2.43	7.26	1.19	6.97
750 (HQC)	5.47	3.28	2.23	3.60

**Table 5:** Summary of stability of QC samples of liensinine under different storage conditions (*n* *=* *3*).

Conditions	Spiked concentration (ng/mL)	Recovery (%)	RSD (%)
12 h RT	0.05	98.10	2.3
	5.0	98.72	4.5
	750	101.11	0.9

Autosampler 24 h	0.05	96.82	4.2
	5.0	100.17	7.7
	750	98.99	3.9

After 3 freeze-thaw cycles	0.05	99.64	0.8
	5.0	103.01	2.4
	750	99.03	0.9

−20°C for one month	0.05	92.57	5.5
	5.0	94.39	9.2
	750	95.12	2.8

**Table 6:** Main pharmacokinetic parameters of liensinine after oral administration at three escalating doses (mean ± SD, *n* *=* *5*).

Pharmacokinetic parameters	Dose (mg/kg)		
	5.0	12.5	25.0
AUC_0–*t*_ (ng h/ml)	60.97 ± 6.91	224.64 ± 60.73	780.56 ± 70.05
AUC_0–∞_ (ng h/ml)	81.92 ± 22.79	279.25 ± 83.77	881.90 ± 106.59
*T*_1/2_ (h)	12.72 ± 6.61	11.05 ± 2.49	11.08 ± 4.25
MRT_0–∞_ (h)	16.95 ± 7.3	13.89 ± 1.55	12..36 ± 2.35
*V*_*z*_ (L/Kg)	64.42 ± 15.84	62.77 ± 24.50	57.57 ± 19.60
CL_z_ (L/h/Kg)	3.89 ± 1.12	3.93 ± 1.20	3.69 ± 0.42
*C*_max_ (*μ*g/L)	6.70 ± 1.32	33.66 ± 12.48	154.74 ± 17.94
*F* (%)	5.98	8.16	12.88

**Table 7:** Results of the main pharmacokinetic parameters of liensinine after intragastric administration of liensinine (5 mg/kg) to rats.

Pharmacokinetic parameters	Mean ± SD (*n* *=* *5*)	
	Intragastric	Intravenous
AUC_0-*t*_ (ng h/ml)	60.97 ± 6.91	1164.09 ± 322.26
AUC_0-∞_ (ng h/ml)	81.92 ± 22.79	1369.09 ± 319.15
*T*_1/2_ (h)	12.72 ± 6.61	9.81 ± 2.39
MRT (h)	16.95 ± 7.3	11.70 ± 2.42
*V* (L/Kg)	64.42 ± 15.84	56.20 ± 24.81
CL (L/h/Kg)	3.89 ± 1.12	3.82 ± 0.91
*C*_max_ (ng/ml)	6.70 ± 1.32	668.4 ± 157.9

## Data Availability

The data used to support the ﬁndings of this study are included within the article.

## Abbreviations

LLOQ: Lower limit of quantification
TLC: Thin-layer chromatography
HSCCC: High-speed countercurrent chromatography
PPT: Protein precipitation
MTBE: Methyl tert-butyl ether
LLE: Liquid-liquid extraction
IS: Internal standard
ACN: Acetonitrile
ESI: Electrospray ionization
MRM: Multiple reaction monitoring
DL: Desolvation line
CE: Collision energy
LOD: Limit of detection
LLE: Liquid-liquid extraction
QC: Quality control.
